# CcpA affects expression of the *groESL *and *dnaK *operons in *Lactobacillus plantarum*

**DOI:** 10.1186/1475-2859-5-35

**Published:** 2006-11-27

**Authors:** Cristiana Castaldo, Rosa A Siciliano, Lidia Muscariello, Rosangela Marasco, Margherita Sacco

**Affiliations:** 1 Dipartimento di Scienze Ambientali, Seconda Università di Napoli, Caserta, Italy; 2 Centro di Spettrometria di Massa Proteomica e Biomolecolare, Istituto di Scienze dell'Alimentazione, CNR, Avellino, Italy; 3 Dipartimento di Scienze della Vita, Seconda Università di Napoli, Caserta, Italy

## Abstract

**Background:**

Lactic acid bacteria (LAB) are widely used in food industry and their growth performance is important for the quality of the fermented product. During industrial processes changes in temperature may represent an environmental stress to be overcome by starters and non-starters LAB. Studies on adaptation to heat shock have shown the involvement of the chaperon system-proteins in various Gram-positive bacteria. The corresponding operons, namely the *dnaK *and *groESL *operons, are controlled by a negative mechanism involving the HrcA repressor protein binding to the *cis *acting element CIRCE.

**Results:**

We studied adaptation to heat shock in the lactic acid bacterium *Lactobacillus plantarum*. The LM3-2 strain, carrying a null mutation in the *ccpA *gene, encoding the catabolite control protein A (CcpA), showed a lower percent of survival to high temperature with respect to the LM3 wild type strain. Among proteins differentially expressed in the two strains, the GroES chaperon was more abundant in the wild type strain compared to the mutant strain under standard growth conditions. Transcriptional studies showed that class I heat shock operons were differentially expressed upon heat shock in both strains. Indeed, the *dnaK *and *groESL *operons were induced about two times more in the LM3 strain compared to the LM3-2 strain. Analysis of the regulatory region of the two operons showed the presence of *cre *sequences, putative binding sites for the CcpA protein.

**Conclusion:**

The *L. plantarum dnaK *and *groESL *operons are characterized by the presence of the *cis *acting sequence CIRCE in the promoter region, suggesting a negative regulation by the HrcA/CIRCE system, which is a common type of control among the class I heat shock operons of Gram-positive bacteria. We found an additional system of regulation, based on a positive control exerted by the CcpA protein, which would interact with *cre *sequences present in the regulatory region of the *dnaK *and *groESL *operons. The absence of the CcpA protein results in a lower induction of the chaperon coding operons, with a consequent lower percent of survival of the LM3-2 mutant strain population with respect to the wild type when challenged with a heat insult.

## Background

Studies on the adaptation to environmental stresses in Lactic acid bacteria (LAB) are of great interest due to the large use of these microorganisms in food industry [1-3]. Indeed the growth performance of the appropriate species of LAB to be used for a fermentation process plays a key role in the development of organoleptic and hygienic quality of the final fermented product. The responses to adverse conditions, which may be encountered during industrial processes, appear therefore to be crucial for fermented food productions. Increasing in temperature during food processing may be one of the stress conditions to be overcome by starters and non-starters LAB. Among the Gram-positive bacteria the response to heat shock has been widely studied in *Bacillus subtilis*, where the expression of over 200 genes is induced at least three folds upon increasing of temperature [4]. These genes have been classified in six classes depending on the different type of transcriptional regulation they undergo [4-6]. Some of these genes belonging to the general stress response regulon (Class II), are controlled by the σ^B ^factor, while others are under the control of the σ^A ^factor and specific regulators. Class I and III are controlled by transcriptional repressors, class IV by transcriptional activators, class V by a two-component signal transduction system, and class VI comprises all the other genes and operons whose regulation system is still unknown [4]. Class I heat shock genes consist of the *dnaK *and *groESL *operons, coding proteins belonging to the two chaperon complexes DnaK-GrpE-DnaJ and GroES-GroEL respectively. Both operons are negatively regulated by the HrcA protein, which specifically binds to the inverted repeat CIRCE (controlling inverted repeat for chaperon expression) under non-stressed conditions. The CIRCE element is composed of a perfect inverted repeat of 9 bp separated by a 9 bp spacer with DNA sequence TTAGCACTC-N9-GAGTGCTAA [4]. A proposed mechanism presumes that the HrcA protein needs the GroE chaperonin system to become active and therefore able to bind the CIRCE element. Upon increasing in temperature, the GroE chaperonin is titrated by non-native proteins arising as a result of the heat shock. Under these conditions GroE is not available for binding with the HrcA protein, which switches from the active (repressor) to the inactive form [4,7]. Chaperones, in their role of assisting protein folding, are key cell components under physiological as well as stress conditions. They have been found in all the investigated organisms belonging to the bacteria and eucarya domains, while few exceptions were found only among species belonging to the archaea domain [8]. In LAB the expression of the chaperon coding operons, classified as Class I heat shock genes in *B. subtilis*, are regulated by the HrcA/CIRCE interaction [9,10]. With the exception of the *dnaK *operon of *Lactococcus lactis*, where two CIRCE elements overlap the promoter sequence, all the other chaperon-coding operons of LAB, whose sequences are available, show a single CIRCE element in the regulatory region [10,11].

In this report we have investigated the expression of class I heat shock genes in *Lactobacillus plantarum*, a lactic acid bacterium widely distributed in different environmental niches including the mammalian gastrointestinal tract [12]. Indeed, *L. plantarum *is considered an important component of the intestinal microbiota of healthy individuals and for its properties is considered a probiotic LAB [1,12]. It is also widely distributed in most fermented products of animal and plant origin either as starter or as NSLAB [13], and more recently it has been used for nutraceutical productions [14]. The availability of its complete genome sequence also allowed the development of metabolic models for improvement of its use as cell factories [15,16]. Studies on the physiological and molecular mechanisms of adaptation of *L. plantarum *to various stresses have been recently reviewed, but little is known about the response of this microorganism to heat shock [17,18]. Gobbetti and co-workers described the increasing expression of the DnaK and GroEL proteins upon adaptation to heat shock in *Lactobacillus plantarum *[17]. We found the involvement of the CcpA protein in the positive regulation of the *dnaK *and *groESL *operons. CcpA has been initially described as the regulatory protein mediating carbon catabolite repression in Gram-positive bacteria; its specific DNA target sequence is named *cre *(catabolite responsive element) [19,20]. It is now well documented the role of CcpA as global regulator involved in the control of various metabolic pathways in different gram-positive bacteria, as well as in the control of specific cell function depending on the bacterial species [21-27].

## Results and discussion

### The CcpA-dependent protein expression

**Figure 1:**
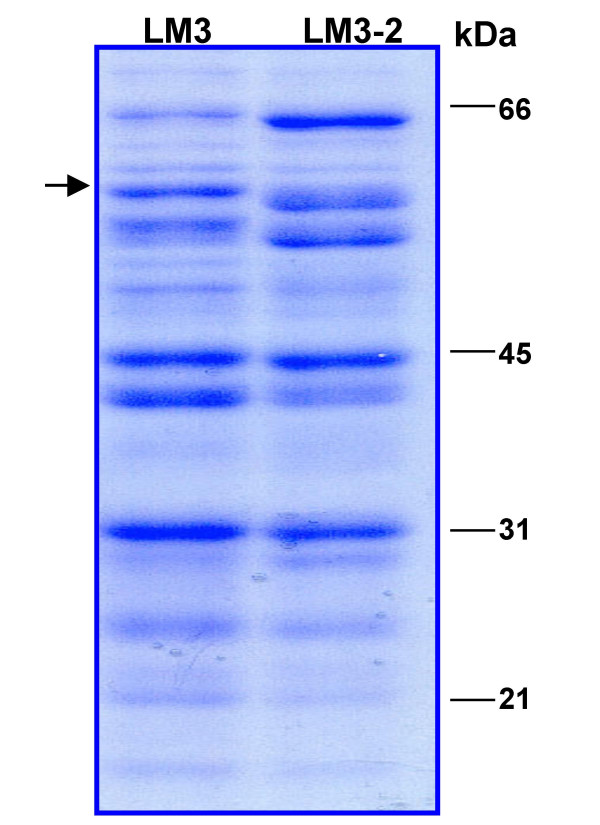
***L. plantarum *LM3 and LM3-2 protein analysis**. SDS-PAGE analysis of the LM3 and LM3-2 cytosolic proteins. The arrow indicates the protein identified as GroEL. Molecular weight markers are indicated.

In a previous work we have isolated the *L. plantarum *LM3-2(*ccpA1*) strain, carrying a null mutation in the *ccpA *gene [28]. We analyzed the electrophoretic pattern of cytosolic proteins extracted from LM3 and LM3-2 cells, grown under standard conditions up to mid-exponential phase (Fig. 1). The presence of differentially expressed proteins in the two strains, together with results of previous studies [28,29] demonstrated the role of CcpA as global regulator in *L. plantarum*. Among the differentially expressed proteins analyzed, one of them, with apparent molecular mass of 60 kDa, was identified by in-gel tryptic digestion and MALDI-TOF (Matrix-assisted laser desorption ionization-time of flight) analysis as the GroEL protein (Fig. 1). Indeed, the GroEL protein appears to be more abundant in the LM3 wild type strain compared to the LM3-2 mutant strain, suggesting the involvement of the CcpA protein in the positive regulation of its expression. In *Bacillus subtilis *the expression of the GroEL and GroES molecular chaperons have been linked to the CcpA protein, but it is interesting to point out that in this model system expression of the two proteins appeared to be under the CcpA negative regulation [30]. The different CcpA-dependent control of the *groESL *operon in the two model systems may have been influenced by the diverse growth condition used.

### CcpA affects heat shock survival

**Figure 2:**
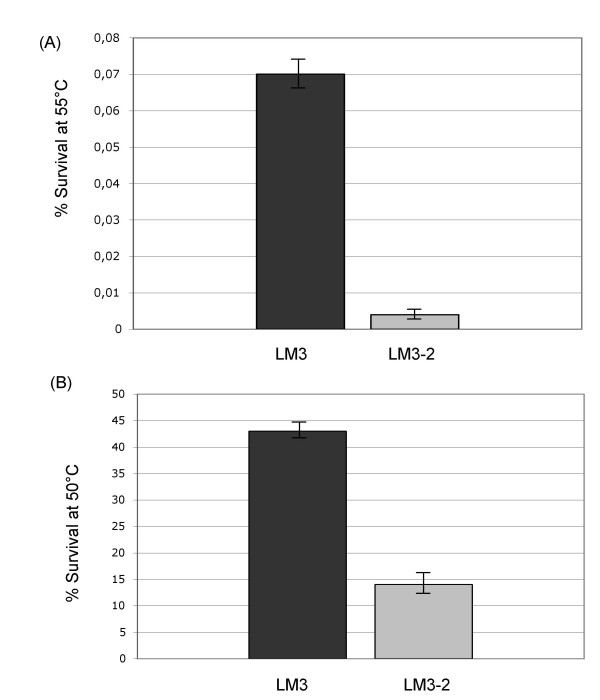
**Heat shock survival**. (A) Heat shock survival of *L. plantarum *LM3 and LM3-2 strains challenged at 55°C; (B) Heat shock survival of *L. plantarum *LM3 and LM3-2 strains challenged at 50°C. Mean of 3 independent experiments, with a standard deviation not exceeding 5%.

CcpA has been found to be involved in various stress responses using different microorganisms as model system. In *Listeria monocytogenes *CcpA is overexpressed under salt stress conditions together with other general metabolism proteins (alanine dehydrogenase, CysK, EF-Tu, Gap, GuaB, PdhA, PdhD), and general stress proteins (Ctc and DnaK) [31]. In *Enterococcus faecalis *CcpA negatively regulates the expression of 13 starvation-inducible proteins [32]. In *Lactococcus lactis *the production of CcpA is induced about three times upon exposure to low temperature [33]. In order to investigate the effect of the *ccpA1 *mutation on the heat shock response in *L. plantarum*, cell cultures of the LM3 (wt) and LM3-2 (*ccpA1*) strains were grown up to exponential phase in MRS with 2% glucose and challenged with high temperature (55°C) for 1 h. The percent of survival for each strain was determined by plating on MRS agar and by comparing the number of live cells after heat shock to the number of live cells before the shock. Under these experimental conditions the percent of survival was extremely low, being 0.07% and 0.004% for LM3 and LM3-2 respectively (Fig. 2A). When the two strains were challenged with the temperature of 50°C for 1 h, the LM3 strain showed 43% survival versus 14% survival of the LM3-2 strain (Fig. 2B). In the first case, the experimental conditions appeared to be very extreme for both strains, still the mutant strain showing a percent of survival 17 fold lower than the wild type strain. When challenged with a less severe heat shock conditions, the LM3-2 strain showed a percent of survival 3 fold lower than the LM3 strain. These results suggest a positive role of CcpA in the heat shock response in *L. plantarum*. The heat shock temperature of 50°C was chosen for further studies.

### Transcriptional analysis of the *groESL *operon

**Figure 3:**
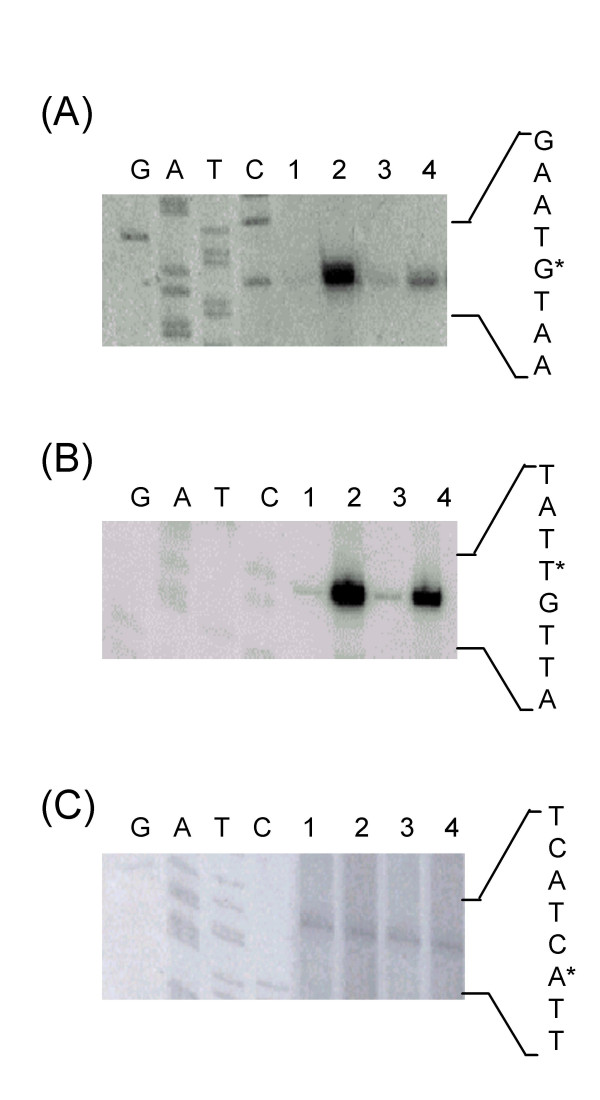
**Transcriptional analysis of class I heat shock operons**. Primer extension analysis of: (A) *groESL*, (B) *dnaK*, and (C) lp_rRNA01 transcripts. Primer extension products were obtained by using oligonucleotides groESL2, hrc2, and sed2, respectively. Total RNA was extracted from: LM3 cells grown at 30°C (lane 1), or after a 15 min heat shock at 50°C (lane 2); LM3-2 cells grown at 30°C (lane 3), or after a 15 min heat shock at 50°C (lane 4). As a reference, sequencing reactions were performed with the same primers.

**Figure 4:**
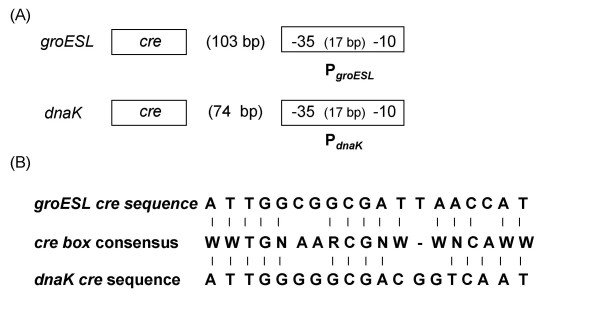
***cre *sequence alignment**. (A) Structure of the *groESL *and *dnaK *promoter regions. The positions of the putative *cre *sequences upstream of the promoters are shown. (B) Comparison of *cre *box consensus sequence [34] with *dnaK *and *groESL *putative *cre *box sequences. Symbols for nucleotides in the consensus sequence: W (A or T); R (A or G); N (A, G, C or T).

Primer extension experiments were performed using total RNA extracted from the LM3 and LM3-2 cell cultures grown up to exponential phase at 30°C and then left at 30°C (control) or challenged at 50°C for 15 minutes. No differences in the *groESL *basal transcription were observed in the two strains when incubated at 30°C; after heat shock at 50°C, instead, a transcriptional activation of the *groESL *operon occurred in both strains, but at a greater extent in the wild type compared to the mutant strain (Fig. 3A). A quantitative analysis by PhosphorImager showed that transcription of the *groESL *operon was induced 12-fold in the LM3 wild type strain (Fig. 3A, lanes 1 and 2), while only 5-fold in LM3-2 mutant strain (Fig. 3A, lanes 3 and 4). As a control, primer extension analysis of transcripts from the lp_rRNA01 locus, transcribing a 16S rRNA, was performed, and a PhosphorImager analysis showed a 1:1 ratio among the 4 samples (Fig. 3C). The sequence analysis of the *groESL *promoter region revealed the presence of the CIRCE element of 9 bp separated by 9 bp spacer, with the DNA sequence TTAGCACTT-N9-GAGTGCTAA, starting four nucleotide residues downstream of the start site of transcription. This putative CIRCE element is characterized by one mismatch with respect to the consensus sequence (9^th ^nucleotide residue position) [6]. Furthermore, the sequence analysis revealed the presence of a putative CcpA-binding site (*cre*), centered at -147.5 with respect to the start point of transcription (Fig. 4A).

### Transcriptional analysis of the *dnaK *operon

On the basis of the observed effect of CcpA on the induction of the *groESL *transcription in *L. plantarum *after exposure to heat shock, we proceeded in the analysis of the expression of the other class I heat shock operon, namely the *dnaK *operon. The *L. plantarum *tetra-cistronic *dnaK *operon consists of the *hrcA *gene, coding for the transcriptional repressor HrcA, followed by the *grpE*, *dnaK *and *dnaJ *genes, coding for the three proteins constituting the DnaK chaperon complex [15]. Primer extension experiments were performed by using total RNA extracted from the LM3 and LM3-2 cell cultures grown up to exponential phase at 30°C and then left at 30°C (control) or challenged at 50°C for 15 minutes (Fig. 3B). The results were similar to those obtained for the transcriptional analysis of the *groESL *operon. No detectable differences were found in the basal expression of the operon at 30°C in the two strains. On the contrary, transcription of the *dnaK *operon was induced 16-fold in the LM3 wild type strain (Fig. 3B, lanes 1 and 2) and only 8-fold in the LM3-2 mutant strain upon exposure to a heat shock (Fig. 3B, lanes 3 and 4), as measured by a quantitative analysis by PhosphorImager. The sequence analysis of the *groESL *promoter region revealed the presence of the CIRCE element of 9 bp separated by 9 bp spacer, with the DNA sequence TTAGCACTC-N9-AAGTGCTAA, starting three nucleotide residues downstream of the start site of transcription. As well as for the *groESL *promoter, this putative CIRCE element is characterized by one mismatch with respect to the consensus sequence (19^th ^nucleotide residue position). A putative CcpA binding site (*cre*) is centered at -117.5 with respect to the start point of transcription (Fig. 4A).

Class I heat shock operons have been shown so far to be negatively regulated by the HrcA/CIRCE system. We show that a positive regulation mediated by the CcpA protein also occurs for full expression of these operons in *L. plantarum*.

### Analysis of *cre *sequences

The *cre *sequence is described as an 18 bp consensus sequence for binding of the CcpA protein in the promoter region of genes and operons subjected to catabolite repression [34]. The two putative *cre *sequences centered at -147.5 and at -117.5 in the promoter region of the *groESL *and *dnaK *operons respectively, contain an additional base with respect to the consensus described by Miwa and coworkers (Fig. 4B) [34]. An additional base also characterizes the *cre1 *element found in the regulatory region of the *ackA *gene of *Bacillus subtilis*, whose expression is controlled by a CcpA-mediated activation mechanism [35]. Nevertheless, the *cre1 *sequence, centered in position -116.5, does not seem to be functional to the CcpA-mediated activation of the operon under the tested conditions, probably due to the presence of the *cre2 *promoter proximal sequence [35].

## Conclusion

We showed that the GroEL protein is differentially expressed in the LM3 strain of *L. plantarum *versus its isogenic LM3-2 (*ccpA1*) mutant strain. Lack of the CcpA protein affects the survival of the LM3-2 cell population after a heat shock insult. Transcription of class I heat shock operons, namely the *groESL *and *dnaK *operons, is positively regulated by CcpA upon exposure to high temperature. Putative *cre *elements were found in the regulatory regions of the *L. plantarum groESL *and *dnaK *operons. To our knowledge this is the first indication that a positive regulation of transcription may also occur for full expression of class I heat shock operons.

## Methods

### Bacterial strains and culture conditions

*L. plantarum *LM3 and LM3-2 [28] were used throughout this study. *L. plantarum *was grown in MRS medium (prepared without carbon source) supplemented with 2% glucose. When needed, chloramphenicol (10 μg/ml) was added to the culture medium.

### Heat shock survival

*L. plantarum *LM3 and LM3-2 cells were grown at 30°C in 100 ml of MRS broth supplemented with 2% glucose, up to exponential phase (OD_595_= 0,7). One ml of each sample was taken, opportunely diluted, and plated on MRS agar with 2% glucose; the cultures were then divided into two equal volumes, named C and H. "H" samples were incubated at 50°C or 55°C for 1 h, whereas "C" samples were left at 30°C. After 1 h, the cells were diluted and plated. After 16 h of incubation at 30°C, the percent of survival was calculated as [(number of live cells after heat shock/initial number of cells) × 100].

### Fractionated cell extracts and SDS-PAGE

LM3 and LM3-2 cells were grown at 30°C in 100 ml of MRS broth supplemented with 2% glucose, up to exponential phase (OD_595 _= 0,7), washed twice with 10 mM Tris-HCl, (pH 7.5), resuspended in 20 ml of the same buffer and French-pressed (3 cycles, 1,500 psi). After two steps of centrifugation (7,500 rpm, 20 min), the supernatants were ultracentrifugated (120,000 × g, 1 h). The supernatants constituted the cytoplasmic fraction. The protein concentration was determined by the Bradford method, using the Protein Assay Kit (BIO-RAD Inc). Ten micrograms of proteins were subjected to SDS-PAGE (10%).

### Protein identification by peptide mass fingerprinting

In-gel digestion was carried out according to Shevchenko and co-workers [36]. Briefly, selected Comassie-stained protein bands were manually excised from gels and destained with 50% acetonitrile (ACN) in 100 mM ammonium bicarbonate, dehydrated in ACN and vacuum-dried in a Speed-Vac centrifuge (Savant). Proteins contained in the gel pieces were treated with 10 mM dithiothreitol in 100 mM ammonium bicarbonate at 56°C for 1 h to reduce disulphide bridges, and alkylation of the cysteine residues was carried out with 50 mM iodoacetamide in 100 mM ammonium bicarbonate at room temperature in the dark for 30 min. Gel bands were dehydrated in ACN and re-swollen in 10 μl of buffer solution (25 mM ammonium bicarbonate pH 8.4) containing 10 ng/μl of trypsin at 4°C for 15 min. The excess of enzymatic solution was removed and 20 μl of buffer solution were added to the gel pieces. Digestion proceeded overnight at 37°C. The obtained peptide mixture (0.5 μl) was mixed with 0.5 μl of a saturated solution of α-cyano-4-hydroxycinnaminic acid (10 mg/ml in 50% ACN containing 25 fmol/μl angiotensin and 125 fmol/μl ACTH (Adrenocorticotropic Hormone fragment 18-39) as internal standards), spotted directly on a MALDI target plate and dried under ambient condition. All mass spectra were generated on a MALDI-TOF mass spectrometer Voyager DE™ PRO (Applied Biosystems), operating in positive-ion reflectron mode. The laser intensity (N_2_, 337 nm) was set just above the ion generation threshold and pulsed every 10 ns. Mass spectra were acquired from each sample in the 700–3500 m/z range, by accumulating 100 laser shots and were calibrated using as internal standards the monoisotopic peaks of angiotensin (m/z 931.5154) and ACTH (m/z 2465.1989). All mass values are reported as monoisotopic masses.

Protein identification was achieved by using the MALDI mass spectral data for database search against the NCBInr database using the MASCOT search algorithm [37]. Parameters for all searches were as follows: bacteria as taxonomic category, trypsin as enzyme, carbamidomethyl as fixed modification for cysteine residues and methionine oxidation as variable modification, one missing cleavage and 30 ppm as mass tolerance for the monoisotopic peptide masses.

### Primer extension analysis

Total RNA from *L. plantarum *cells grown to mid-exponential phase on MRS medium supplemented with 2% glucose was isolated as described [28]. Primer extension products of *groESL*, *dnaK*, and lp_rRNA01 transcripts were obtained using oligonucleotides groESL2 (5'-CCGCTTTGGGGTTTTCCTTAGCG-3'), hrc2 (5'-GGATCCAACTGGATTACCGCCC-3'), and sed2 (5'-GGTGTTATCCCCCGC-3'), respectively. The experiments were performed as previously described [28].

## Competing interests

The author(s) declare that they have no competing financial interests.

## Authors' contributions

CC performed most of the experiments; RAS performed the MALDI-TOF analysis; LM performed the heat shock survival experiments; RM contributed to experiment design and discussion; MS contributed in discussions during the work and in the preparation of the manuscript. All authors read and approved the final manuscript.
